# Playing hide and seek with repeats in local and global de novo transcriptome assembly of short RNA-seq reads

**DOI:** 10.1186/s13015-017-0091-2

**Published:** 2017-02-22

**Authors:** Leandro Lima, Blerina Sinaimeri, Gustavo Sacomoto, Helene Lopez-Maestre, Camille Marchet, Vincent Miele, Marie-France Sagot, Vincent Lacroix

**Affiliations:** 1 Inria Grenoble, 655, Avenue de l’Europe, 38334 Montbonnot, France; 2 CNRS, UMR5558, Université Claude Bernard Lyon 1, 43, Boulevard du 11 Novembre 1918, 69622 Villeurbanne, France; 3 IRISA Inria Rennes Bretagne Atlantique; GenScale Team, Université Rennes 1, 263, Avenue Général Leclerc, 35042 Rennes, France

**Keywords:** Transcriptome assembly, RNA-seq, Repeats, Alternative splicing, Formal model for representing repeats, Enumeration algorithm, De Bruijn graph topology, Assembly evaluation

## Abstract

**Background:**

The main challenge in de novo genome assembly of DNA-seq data is certainly to deal with repeats that are longer than the reads. In de novo transcriptome assembly of RNA-seq reads, on the other hand, this problem has been underestimated so far. Even though we have fewer and shorter repeated sequences in transcriptomics, they do create ambiguities and confuse assemblers if not addressed properly. Most transcriptome assemblers of short reads are based on de Bruijn graphs (DBG) and have no clear and explicit model for repeats in RNA-seq data, relying instead on heuristics to deal with them.

**Results:**

The results of this work are threefold. First, we introduce a formal model for representing high copy-number and low-divergence repeats in RNA-seq data and exploit its properties to infer a combinatorial characteristic of repeat-associated subgraphs. We show that the problem of identifying such subgraphs in a DBG is NP-complete. Second, we show that in the specific case of local assembly of alternative splicing (AS) events, we can *implicitly* avoid such subgraphs, and we present an efficient algorithm to enumerate AS events that are not included in repeats. Using simulated data, we show that this strategy is significantly more sensitive and precise than the previous version of KisSplice (Sacomoto et al. in WABI, pp 99–111, 1), Trinity (Grabherr et al. in Nat Biotechnol 29(7):644–652, 2), and Oases (Schulz et al. in Bioinformatics 28(8):1086–1092, 3), for the specific task of calling AS events. Third, we turn our focus to full-length transcriptome assembly, and we show that exploring the topology of DBGs can improve de novo transcriptome evaluation methods. Based on the observation that repeats create complicated regions in a DBG, and when assemblers try to traverse these regions, they can infer erroneous transcripts, we propose a measure to flag transcripts traversing such troublesome regions, thereby giving a confidence level for each transcript. The originality of our work when compared to other transcriptome evaluation methods is that we use only the topology of the DBG, and not read nor coverage information. We show that our simple method gives better results than Rsem-Eval (Li et al. in Genome Biol 15(12):553, 4) and TransRate (Smith-Unna et al. in Genome Res 26(8):1134–1144, 5) on both real and simulated datasets for detecting chimeras, and therefore is able to capture assembly errors missed by these methods.

## Background

Transcriptomes can now be studied through sequencing. However, in the absence of a reference genome, de novo assembly remains a challenging task. The main difficulty certainly comes from the fact that sequencing reads are short, and repeated sequences within transcriptomes could be longer than the reads. This short read/long repeat issue is of course not specific to transcriptome sequencing. It is an old problem that has been around since the first algorithms for genome assembly. Even though the problems repeats cause in both contexts are similar, they have also some characteristics that are specific to each. In genome assembly, repeats tend to be longer and present in more copies. In transcriptome assembly, repeats are located within genes and tend to be shorter and in fewer copies. However, in this last case, coverage cannot be applied to discriminate contigs that correspond to repeats, as it can be in genomics by using e.g. Myers’ A-statistics [6, 7], since the coverage of a gene does not only reflect its copy-number in the genome, but also and mostly its expression level. Some genes are highly expressed and therefore highly covered, while most genes are poorly expressed and therefore poorly covered. Such specificities complicate the application of a genomic repeat-solving strategy to the transcriptomic context.

Initially, it was thought that repeats would not be a major issue in RNA-seq, since they are mostly in introns and intergenic regions. However, the truth is that many regions which are thought to be intergenic are transcribed [8] and introns are not always already spliced out when mRNA is collected to be sequenced [9]. Repeats, especially transposable elements, are therefore very present in real samples and cause major problems in transcriptome assembly, if not addressed properly.

Most, if not all current short-read transcriptome assemblers are based on de Bruijn graphs. Among the best known are Oases [3], Trinity [2], and to a lesser degree Trans-Abyss [10] and IDBA-tran [11]. Common to all of them is the lack of a clear and explicit model for repeats in RNA-seq data. Heuristics are thus used to try and cope efficiently with repeats. For instance, in Oases short vertices are thought to correspond to repeats and are therefore not used for assembling genes. They are added in a second step, which hopefully causes genes sharing repeats not to be assembled together. In Trinity, there is no attempt to deal with repeats by explicitly modelling them. The first module of Trinity, Inchworm, will try and assemble the most covered contig which hopefully corresponds to the most abundant alternative transcript. Then alternative exons are glued to this major transcript to form a splicing graph. The last step is to enumerate all alternative transcripts. If repeats are present, their high coverage may be interpreted as a highly expressed link between two unrelated transcripts. Overall, assembled transcripts may be chimeric or spliced into many sub-transcripts.

In the method we had previously developed, KisSplice, which is a local transcriptome assembler [12], repeats are less problematic since the goal is not to assemble full-length transcripts. KisSplice instead aims at finding variants in transcriptomes (SNPs, indels and alternative splicings). However, as we reported in [12], KisSplice was not able to deal with large portions of a de Bruijn graph containing subgraphs associated to highly repeated sequences, e.g. transposable elements, the so-called complex Biconnected Components.

Here, we try and achieve three goals: (1) give a clear formalisation of the notion of repeats with high copy-number in RNA-seq data, (2) apply it on local transcriptome assembly by giving a practical way to enumerate bubbles that are lost because of such repeats, and (3) apply it on global transcriptome assembly by showing that the topology of the subgraph around a transcript can give some hints about its confidence level. Recall that we are in a de novo context, so we assume that neither a reference genome/transcriptome nor a database of known repeats, e.g. RepBase [13], are available.

First, we formally introduce a model for representing high copy-number repeats and exploit its properties to infer that repeat-associated subgraphs in a de Bruijn graph contain few compressible arcs. However, we show that the problem of identifying, in a de Bruijn graph, a subgraph corresponding to repeats according to such characterisation is NP-complete. A polynomial time algorithm is therefore unlikely to exist.

Second, we show that in the specific case of a local assembly of alternative splicing (AS) events, by using a strategy based on the compressible-arc characterization, we can *implicitly* avoid such subgraphs. More precisely, it is possible to find the structures (i.e. bubbles) corresponding to AS events in a de Bruijn graph that are not contained in a repeat-associated subgraph (see Fig. 3 for an example). While there has been great efforts in the literature to solve repeats, there has been almost no exploration on how to avoid them. This is explained by the fact that most efforts in assembly concentrate on full-length genome and transcriptome assembly, in which avoiding repeats is not an option, and the performance of an assembler can be narrowed down to how well it solves repeats. However, in our case, repeat-avoidance can be an effective technique. Indeed, this fact was confirmed by our experiments, where using human simulated RNA-seq data, we show that the new algorithm improves significantly the sensitivity of KisSplice, while also improving its precision. We further compared our algorithm to two of the best transcriptome assemblers, namely Trinity [2] and Oases [3], in the specific task of calling AS events, and we show that our algorithm is more sensitive than both tools, while also being more precise. In addition, our results show that the advantage of using the new algorithm proposed in this work is more evident when the input data contains high pre-mRNA content or the AS events of interest stem from highly-expressed genes. Moreover, we give an indication of the usefulness of our method on real data.

Third, we show that the method described can also be applied in the context of full-length transcriptome assembly. We introduce a measure based on the proposed model to identify low-confidence transcripts, which are the ones that traverse complex regions in the de Bruijn Graph. Within these complex parts of the graph generated by repeats, any assembler will have to choose the “right” path(s) among the many present. This choice is not simple and may lead to incorrect solutions (e.g. chimeric or truncated transcripts). It is therefore important to be able to identify the transcripts coming from such complex regions in order to know that the solution presented is not the only one, and furthermore may not be the right one. We compared our measure against two state-of-the-art methods for de novo transcriptome evaluation, namely Rsem-Eval [4] and TransRate [5], for the specific task of identifying chimeric transcripts in both real and simulated datasets. We show that our measure provides good results despite the fact that it uses only the graph topology, and not coverage, nor read information. The results obtained thus suggest that exploring the topology of the subgraph around a transcript, an information that is currently disregarded by transcriptome evaluation methods, can be useful to infer some of the transcript’s properties, such as confidence level, quality, assembly hardness, etc. Therefore, our measure can improve the state-of-the-art methods for de novo transcriptome evaluation, since it is able to capture assembly errors missed by these tools.

### Preliminaries

Let $\Sigma$ be an alphabet of fixed size $\sigma$. Here we always assume $\Sigma =\{A,C,T,G\}$. Given a sequence (string) $s \in \Sigma ^*$, let |*s*| denote its length, *s*[*i*] the *i*th element of *s*, and *s*[*i*, *j*] the substring $s[i] s[i+1] \ldots s[j]$ for any $1 \le i<j \le |s|$.

A *k-mer* is a sequence $s \in \Sigma ^k$. Given an integer *k* and a set *S* of sequences each of length $n \ge k$, we define *span*(*S*, *k*) as the set of all distinct *k*-mers that appear as a substring in *S*.

#### **Definition 1**

Given a set of sequences (reads) $R \subseteq \Sigma ^*$ and an integer *k*, we define the directed de Bruijn graph $G_k(R)=(V,A)$ where $V=span(R,k)$ and $(u,v) \in A$ if and only if $u[2,k]=v[1,k-1]$.

Given a directed graph $G = (V,A)$ and a vertex $v \in V$, we denote its *out-neighbourhood* (resp. *in-neighbourhood*) by $N^+(v)=\{ u \in V \mid (v,u) \in A \}$ (resp. $N^-(v)=\{ u \in V \mid (u,v) \in A \}$), and its *out-degree* (resp. *in-degree* by $d^+(v)=|N^+(v)|$ ($d^-(v)=|N^-(v)|$). A (simple) *path* $\pi = s \leadsto t$ in *G* is a sequence of distinct vertices $s = v_0, \ldots , v_l = t$ such that, for each $0 \le i < l$, $(v_i, v_{i+1})$ is an arc of *G*. If the graph is weighted, i.e. there is a function $w : A \rightarrow Q_{\ge 0}$ associating a weight to every arc in the graph, then the *length* of a path $\pi$ is the sum of the weights of the traversed arcs, and is denoted by $|\pi |$.

**Fig. 1:**
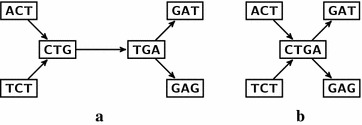
Example of compressible arc in a de Bruijn graph. **a** The arc (*CTG*, *TGA*) is the only compressible arc in the given de Bruijn graph ($k=3$). **b** The corresponding compressed de Bruijn graph

An arc $(u,v) \in A$ is called *compressible* if $d^+(u)=1$ and $d^-(v)=1$. The intuition behind this definition comes from the fact that every path passing through *u* should also pass through *v*. It should therefore be possible to “compress” or contract this arc without losing any information. Note that the compressed de Bruijn graph [2, 3] commonly used by transcriptomic assemblers is obtained from a de Bruijn graph by replacing, for each compressible arc (*u*, *v*), the vertices *u*, *v* by a new vertex *x*, where $N^-(x) = N^-(u)$, $N^+(x) = N^+(v)$ and the label is the concatenation of the *k*-mer of *u* and the *k*-mer of *v* without the overlapping part (see Fig. 1).

## Repeats in de Bruijn graphs

Given a de Bruijn graph $G_k(R)$ generated by a set of reads *R* for which we do not have any prior information, our goal is to identify whether there are subgraphs of $G_k(R)$ that correspond each to a set of high copy-number repeats in *R*. To this end, we identify and then exploit some of the topological properties of the subgraphs that are induced by repeats. Starting with a formal model for representing repeats with high-copy number, we show that the number of compressible arcs, which we denote by $\gamma$, is a relevant parameter for such a characterisation. This parameter will play an important role in the algorithm of “Bubbles “drowned” in repeats” section. However, we also prove that, for an arbitrary de Bruijn graph, identifying a subgraph $G'$ with bounded $\gamma (G')$ is NP-complete.

### Simple uniform model for repeats

We now present the model we adopted for representing high copy-number repeats, e.g. transposable elements, in a genome or transcriptome. First, we would like to clarify that our model is a simple one and, as such, should be seen as only a first approximation, yet realistic enough, of what may happen in reality. We consider here that sequencing errors can be successfully removed. Indeed, there are several techniques to remove the big majority of the sequencing errors in RNA-seq data. In KisSplice, for example, we prune the de Bruijn graph using an absolute and a relative cut-off based on the *k*-mer coverage. The absolute cut-off enables us to remove sequencing errors in general, and the relative one is tailored to deal with highly-expressed genes (more details can be found in [14]). Furthermore, while we realise that there is room for improvement, in practice, the sequencing-error-removal procedure in KisSplice seems to be effective, as most sequencing errors are removed at the expense of losing some rare genomic variants [14].

Basically, our model consists of several “similar” sequences, each generated by uniformly mutating a fixed initial sequence. In particular, it enables to model well recent invasions of transposable elements which often involve high copy-number and low divergence rate (i.e. divergence from their consensus sequence). Consider indeed as an example the recent subfamilies AluYa5 and AluYb8 with 2640 and 1852 copies respectively, which both present a divergence rate below $1\%$ [15] (see [16] for other subfamilies with high copy-number and low divergence).

The model is as follows. First, due to mutations, the sequences $s_1, \ldots , s_m$ that represent the repeats are not identical. However, provided that the number of such mutations is not high (otherwise the concept of repeats would not apply), the repeats are considered “similar” in the sense of having a small pairwise Hamming distance between them. We recall that, given two equal length sequences $s$ and $s'$ in $\Sigma ^n$, their *Hamming distance*, denoted by $d_H(s,s')$, is the number of positions *i* for which $s[i] \ne s'[i]$. Indels are thus not considered in this model.

**Fig. 2:**
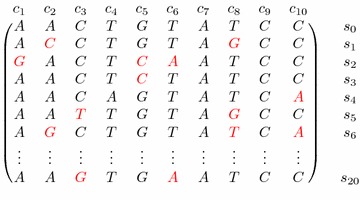
An example of a set of repeats *S*(20, 10, 0.1)

The model has then the following parameters: $\Sigma$, the length *n* of the repeat, the number *m* of copies of the repeat, an integer *k* (for the length of the *k*-mers considered), and the mutation rate, $\alpha$, i.e. the probability that a mutation happens in a particular position. The sequences $s_1, \ldots , s_m$ are then generated by the following process. We first choose uniformly at random a sequence $s_0 \in \Sigma ^n$. At step $i \le m$, we create a sequence $s_i$ as follows: for each position *j*, $s_i[j]=s_0[j]$ with probability $1-\alpha$, whereas with probability $\alpha$ a value different from *s*[*j*] is chosen uniformly at random for $s_i[j]$. We repeat the whole process *m* times and thus create a set $S(m,n,\alpha )$ of *m* such sequences from $s_0$ (see Fig. 2 for a small example). The generated sequences thus have an expected Hamming distance of $\alpha n$ from $s_0$.

### Topological characterisation of the subgraphs generated by repeats

Given a de Bruijn graph $G_k(R)$, if *a* is a compressible arc labelled by the sequence $s=s_1 \ldots s_{k+1}$ then, by definition, *a* is the only outgoing arc of the vertex labelled by the sequence *s*[1, *k*] and the only incoming arc of the vertex labelled by the sequence $s[2,k+1]$. Hence the $(k-1)$-mer *s*[2, *k*] appears as a substring in *R*, always preceded by the symbol *s*[1] and followed by the symbol $s[k+1]$. We refer to such $(k-1)$-mers as being *boundary rigid*. It is not difficult to see that the set of compressible arcs in a de Bruijn graph $G_k(R)$ stands in a one-to-one correspondence with the set of boundary rigid $(k-1)$-mers in *R*.

We now calculate and compare among them the expected number of compressible arcs in $G=G_k(R)$ when *R* corresponds to a set of sequences that are generated: (1) uniformly at random, and (2) according to our model. We show that $\gamma$ is “small” in the cases where the induced graph corresponds to similar sequences, which provides evidence for the relevance of this parameter.

#### **Claim 1**

*Let R* *be a set of m sequences randomly chosen from* $\Sigma ^n$.* Then the expected number of compressible arcs in* $G_k(R)$ *is* $\Theta (mn)$.

#### *Proof*

The probability that a sequence of length $k-1$ occurs in a fixed position in a randomly chosen sequence of length *n* is $(1/4)^{k-1}$. Thus the expected number of appearances of a sequence of length $k-1$ in a set of *m* randomly chosen sequences of length *n* is given by $m(n-k+2) (1/4)^{k-1}$. If $m(n-k+2) \le 4^{k-1}$, then this value is upper bounded by 1, and all the sequences of length $k-1$ are expected to be boundary rigid (as a sequence is expected to appear once). The claim follows by observing that there are $m(n-k+2)$ different $(k-1)$-mers.□

We consider now $\gamma (G_k(R))$ for $R=S(m,n,\alpha )$. We upper bound the expected number of compressible arcs by upper bounding the number of boundary rigid $(k-1)$-mers.

#### **Theorem 1**

*Given integers* *k*, *n*, *m* *with* $k<n$ *and a real number* $0\le \alpha \le 3/4$,* the de Bruijn graph* $G_k(S(m,n,\alpha ))$ *has* *o*(*nm*)* expected compressible arcs.*

#### *Proof*

Let $s_0$ be a sequence chosen randomly from $\Sigma ^n$. Let $S(m,n,\alpha )$ be the set $\{s_1, \ldots , s_m\}$ of *m* repeats generated according to our model starting from $s_0$. Consider now the de Bruijn graph $G=G_k(S(m,n,\alpha ))$. Recall that the number of compressible arcs in this graph is equal to the number of boundary rigid $(k-1)$-mers in $S(m,n,\alpha )$. Let *X* be a random variable representing the number of boundary rigid $(k-1)$-mers in *G*. Consider the repeats in $S(m,n,\alpha )$ in a matrix-like ordering as in Fig. 2 and observe that the mutations from one column to another are independent. Due to the symmetry and the linearity of the expectation, *E*[*X*] is given by $m(n-k+2)$ (the total number of $(k-1)$-mers) multiplied by the probability that a given $(k-1)$-mer is boundary rigid.

The probability that the $(k-1)$-mer $\hat{s}=s[i,i+k-2]$ is boundary rigid clearly depends on the distance from the starting sequence $\hat{s}_0=s_0[i,i+k-2]$. Let *d* be the distance $d_H(\hat{s} ,\hat{s}_0)$.

Observe that if the $(k-1)$-mer $s[i] \ldots s[i+k-2]$ is not boundary rigid then there exists a sequence *y* in $S(m,n,\alpha )$ such that $y[j]= s[j]$ for all $i \le j \le i+k-2$ and either $y[i+k-1] \ne s[i+k-1]$ or $y[i-1] \ne s[i-1]$. It is not difficult to see that the probability that this happens is lower bounded by $(2\alpha -4/3 \alpha ^2) (1-\alpha )^{k-1-d} (\alpha /3)^d$. Hence we have:

$$\begin{aligned} Pr[\hat{s} \text { is boundary rigid} | d_H(\hat{s} ,\hat{s}_0)=d ] \le \Bigl ( 1- (2\alpha -4/3 \alpha ^2) (1-\alpha )^{k-1-d} (\alpha /3)^d\Bigr )^{m-1}. \end{aligned}$$

By approximating the above expression we therefore have that:

1 $$\begin{aligned} \displaystyle E[X]&\le (n-k-1)m \sum _{d=0}^{k-1} Pr[\hat{s} \text { is boundary rigid} | d_H(\hat{s} ,\hat{s}_0)=d ] \\ \nonumber&\le (n-k-1) m e^{- (m-1)(2\alpha -4/3 \alpha ^2)/(\frac{\alpha }{3})^{k-1}}. \end{aligned}$$

For a sufficiently large number of copies $$\left({\text{e.g}}. m=\left( {\begin{array}{c}k\\ \alpha k\end{array}}\right)\right)$$ and using the fact that $$\left( {\begin{array}{c}k\\ \alpha k\end{array}}\right) \ge (1/ \alpha )^{\alpha k}$$, we have that *E*[*X*] is *o*(*mn*). This concludes the proof.□

The previous result shows that the number of compressible arcs is a good parameter for characterising a repeat-associated subgraph.

### Identifying a repeat-associated subgraph

As we showed, a subgraph due to repeated elements has a distinctive feature: it contains few compressible arcs. Based on this, a natural formulation to the repeat identification problem in RNA-seq data is to search for large enough subgraphs that do not contain many compressible arcs. This is formally stated in Problem 1. In order to disregard trivial solutions, it is necessary to require a large enough *connected* subgraph, otherwise any set of disconnected vertices or any small subgraph would be a solution. Unfortunately, we show that this problem is NP-complete, so an efficient algorithm for the repeat identification problem based on this formulation is unlikely.

#### Problem 1

[Repeat Subgraph] *INSTANCE:* A directed graph *G* and two positive integers *m*, *t*.

*DECIDE:* If there exists a connected subgraph $G'=(V', E')$, with $|V'| \ge m$ and having at most *t* compressible arcs.

In Theorem 2, we prove that this problem is NP-complete for all directed graphs with (total) degree, i.e. sum of in and out-degree bounded by 3. The reduction is from the Steiner tree problem which requires finding a minimum weight subgraph spanning a given subset of vertices. It remains NP-hard even when all arc weights are 1 or 2 (see [17]). This version of the problem is denoted by STEINER(1, 2). More formally, given a complete undirected graph $G = (V,E)$ with arc weights in $\{1,2\}$, a set of *terminal* vertices $N \subseteq V$ and an integer *B*, it is NP-complete to decide if there exists a subgraph of *G* spanning *N* with weight at most *B*, i.e. a connected subgraph of *G* containing all vertices of *N*.

We specify next a family of directed graphs that we use in the reduction. Given an integer *x*, we define the directed graph *R*(*x*) as a cycle on 2*x* vertices numbered in a clockwise order and where the arcs have alternating directions, i.e. for any $i \le x$, $(v_{2i},v_{2i+1})$ is an arc. Observe that in *R*(*x*), all vertices in even positions, i.e. all vertices $v_{2i}$, have out-degree 2 and in-degree 0, while all vertices $v_{2i+1}$ have out-degree 0 and in-degree 2. Clearly, none of the arcs of *R*(*x*) is compressible.

#### **Theorem 2**

*The Repeat Subgraph Problem is NP-complete even for directed graphs with degree bounded by* d, for any $d \ge 3$.

#### *Proof*

Given a complete graph $G = (V,E)$, a set of terminal vertices *N* and an upper bound *B*, i.e. an instance of STEINER(1, 2), we transform it into an instance of the *Repeat Subgraph Problem* for a graph $G'$ with degree bounded by 3. Let us first build the graph $G' = (V', E')$. For each vertex *v* in $V \setminus N$, add a corresponding subgraph $r(v) = R(|V|)$ in $G'$ and for each vertex *v* in *N*, add a corresponding subgraph $r(v) = R(|E|+|V|^2 + 1)$ in $G'$. For each arc (*u*, *v*) in *E* with weight $w \in \{1,2\}$, add a simple directed path composed by *w* compressible arcs connecting *r*(*u*) to *r*(*v*) in $G'$; these are the subgraphs corresponding to *u* and *v*. The first vertex of the path should be in a sink of *r*(*u*) and the last vertex in a source of *r*(*v*). By construction, there are at least |*V*| vertices with in-degree 2 and out-degree 0 (sink) and |*V*| vertices with out-degree 2 and in-degree 0 (source) in both *r*(*v*) and *r*(*u*). It is clear that $G'$ has degree bounded by 3. Moreover, the size of $G'$ is polynomial in the size of *G* and it can be constructed in polynomial time.

In this way, the graph $G'$ has one subgraph for each vertex of *G* and a path with one or two (depending on the weight of the corresponding arc) compressible arcs for each arc of *G*. Thus, there exists a subgraph spanning *N* in *G* with weight at most *B* if and only if there exists a subgraph in $G'$ with at least $m =2|N| + 2|E||N| + 2|V|^2|N|$ vertices and at most $t = |B|$ compressible arcs. This follows from the fact that any subgraph of $G'$ with at least *m* vertices necessarily contains all the subgraphs *r*(*v*), where $v \in N$, since the number of vertices in all *r*(*v*), with $v \in V\setminus N$, is at most $|E| + 2|V|^2$ and the only compressible arcs of $G'$ are in the paths corresponding to the arcs of *G*.□

We can obtain the same result for the specific case of subgraphs of de Bruijn graphs. The reduction is more technical but follows similarly.

#### **Theorem 3**

*The Repeat Subgraph Problem is NP-complete even for subgraphs of de Bruijn graphs on* $|\Sigma | = 4$ *symbols*.

## Bubbles “drowned” in repeats

In the previous section, we showed that an efficient algorithm to *directly* identify the subgraphs of a de Bruijn graph corresponding to repeated elements according to our model (i.e. containing few compressible arcs), is unlikely to exist since the problem is NP-complete. However, in this section we show that in the specific case of a local assembly of alternative splicing (AS) events based on the compressible-arc characterisation of “Topological characterisation of the subgraphs generated by repeats” section, we can *implicitly* avoid such subgraphs. More precisely, it is possible to find the structures (i.e. bubbles) corresponding to AS events in a de Bruijn graph that are not contained in a repeat-associated subgraph, thus answering to the main open question of [12].

**Fig. 3:**
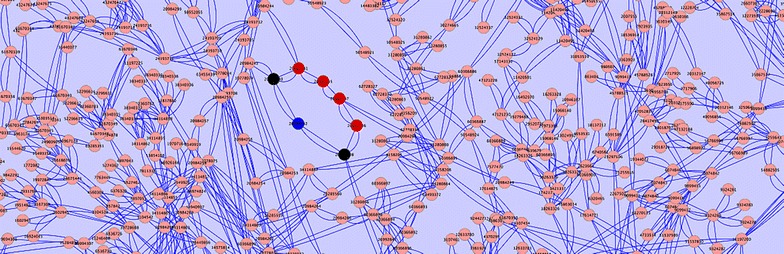
An alternative splicing event in the SCN5A gene (human) [22] trapped inside a complex region, likely containing repeat-associated subgraphs, in a de Bruijn graph. The alternative isoforms correspond to a pair of paths shown in *red* and *blue*

KisSplice [12] is a method for de novo calling of AS events through the enumeration of so-called *bubbles*, that correspond to pairs of vertex-disjoint paths in a de Bruijn graph. The bubble enumeration algorithm proposed in [12] was later improved in [1]. However, even the improved algorithm is not able to enumerate all bubbles corresponding to AS events in a de Bruijn graph. There are certain complex regions in the graph, likely containing repeat-associated subgraphs but also real AS events [12], where both algorithms take a huge amount of time. Figure 3 shows an example of a complex region with a bubble corresponding to an AS event. In practice, the enumeration is halted after a given timeout. The bubbles *drowned* (or trapped) inside these regions are thus missed by KisSplice.

In “Repeats in de Bruijn graphs” section, the repeat-associated subgraphs are characterised by the presence of few compressible arcs. This suggests that in order to avoid repeat-associated subgraphs, we should restrict the search to bubbles containing many compressible arcs. Equivalently, in a compressed de Bruijn graph (see “Preliminaries” section), we should restrict the search to bubbles with few branching vertices. We recall that a *branching vertex* is a vertex of in-degree or out-degree strictly at least 2. Indeed, in a compressed de Bruijn graph, given a fixed sequence length, the number of branching vertices in a path is inversely proportional to the number of compressible arcs of the corresponding path in the non-compressed de Bruijn graph. We thus modify the definition of $(s,t,\alpha _1,\alpha _2)$-bubbles in compressed de Bruijn graphs (Def. 1 in [1]) by adding the extra constraint that each path should have at most *b* branching vertices.

### **Definition 2**

Given a weighted directed graph $G = (V,E)$ and two vertices $s,t \in V$, an $(s,t,\alpha _1,\alpha _2,b)$-bubble is a pair of vertex-disjoint *st*-paths $\pi _1$, $\pi _2$ with lengths bounded by $\alpha _1,\alpha _2$, each containing at most *b* branching vertices.

By restricting the search to bubbles with few branching vertices, we are able to enumerate them in complex regions implicitly avoiding repeat-associated subgraphs. Indeed, in  “Experimental results” section we show that by considering bubbles with at most *b* branching vertices in KisSplice, we increase both its sensitivity and precision. This supports our claim that by focusing on $(s,t,\alpha _1,\alpha _2,b)$-bubbles, we avoid repeat-associated subgraphs and recover at least part of the bubbles trapped in complex regions.

### Enumerating bubbles avoiding repeats

In this section, we modify the algorithm of [1] to enumerate all bubbles with at most *b* branching vertices in each path. Given a weighted directed graph $G = (V,E)$ and a vertex $s \in V$, let $\mathcal {B}_s(G)$ denote the set of $(s,*,\alpha _1,\alpha _2,b)$-bubbles of *G*. The algorithm recursively partitions the solution space $\mathcal {B}_s(G)$ at every call until the considered subspace is a singleton (contains only one solution), and in that case it outputs the corresponding solution. In order to avoid unnecessary recursive calls, it maintains the invariant that the current partition contains at least one solution. The algorithm proceeds as follows.

#### *Invariant*

At a generic recursive step on vertices $u_1,u_2$ (initially, $u_1 = u_2 =s$), let $\pi _1 = s \leadsto u_1, \pi _2 = s \leadsto u_2$ be the paths discovered so far (initially, $\pi _1,\pi _2$ are empty). Let $G'$ be the current graph (initially, $G' := G$). More precisely, $G'$ is defined as follows: remove from *G* all the vertices in $\pi _1$ and $\pi _2$ but $u_1$ and $u_2$. Moreover, we also maintain the following invariant (INV): there exists at least one pair of paths $\bar{\pi }_1$ and $\bar{\pi }_2$ in $G'$ that extend $\pi _1$ and $\pi _2$ so that $\pi _1 \cdot \bar{\pi }_1$ and $\pi _2 \cdot \bar{\pi }_2$ belong to $\mathcal {B}_s(G)$.

#### *Base case*

When $u_1 = u_2 = u$, output the $(s,u,\alpha _1,\alpha _2,b)$-bubble given by $\pi _1$ and $\pi _2$.

#### *Recursive rule*

Let $\mathcal {B}_{s}(\pi _1,\pi _2, G')$ denote the set of $(s,*,\alpha _1,\alpha _2,b)$-bubbles to be listed by the current recursive call, i.e. the subset of $\mathcal {B}_s(G)$ with prefixes $\pi _1, \pi _2$. It is the union of the following disjoint sets:

- The bubbles of $\mathcal {B}_{s}(\pi _1,\pi _2, G')$ that use *e*, for each arc $e = (u_1,v)$ outgoing from $u_1$, that is $\mathcal {B}_{s}(\pi _1 \cdot e, \pi _2, G' - u_1)$, where $G'-u_1$ is the subgraph of $G'$ after the removal of $u_1$ and all its incident arcs.
- The bubbles that do not use any arc from $u_1$, that is $\mathcal {B}_{s}(\pi _1,\pi _2, G'')$, where $G''$ is the subgraph of $G'$ after the removal of all arcs outgoing from $u_1$.

The same holds for $u_2$ instead of $u_1$.

In order to maintain the invariant (INV), we only perform the recursive calls when $\mathcal {B}_{s}(\pi _1 \cdot e, \pi _2, G' - u)$ or $\mathcal {B}_{s}(\pi _1,\pi _2, G'')$ are non-empty. In both cases, we have to decide if there exists a pair of (internally) vertex-disjoint paths $\bar{\pi }_1 = u_1 \leadsto t_1$ and $\bar{\pi }_2 = u_2 \leadsto t_2$, such that $|\bar{\pi }_1| \le \alpha _1'$, $|\bar{\pi }_2| \le \alpha _2'$, and $\bar{\pi }_1,\bar{\pi }_2$ have at most $b_1,b_2$ branching vertices, respectively. Since both the length and the number of branching vertices are monotonic properties, i.e. both are smaller for a prefix instead of for the full path, we can drop the vertex-disjoint condition. Indeed, let $\bar{\pi }_1$ and $\bar{\pi }_2$ be a pair of paths satisfying all conditions but the vertex-disjoint one. The prefixes $\bar{\pi }^*_1 = u_1 \leadsto t^*$ and $\bar{\pi }^*_2 = u_2 \leadsto t^*$, where $t^*$ is the first intersection of the paths, satisfy all conditions and are internally vertex-disjoint.

Moreover, using a dynamic programming algorithm, we can obtain the following result.

#### **Lemma 1**

*Given a non-negatively weighted directed graph* $G = (V,E)$ *and a source* $s \in V$,* we can compute the shortest paths from* *s* *using at most* *b* *branching vertices in* *O*(*b*|*E*|)* time*.

#### *Proof*

Let $d[\beta , t]$ denote the distance from *s* to *t* using at most $\beta$ branching vertices (*s* is never counted as a branching vertex, even if it is branching). The recurrence to calculate $d[\beta ,t]$, for $0 \le \beta \le b$ and $t \in V$ is:

*Initialisation step:*

$$\begin{aligned} \begin{array}{ll} d[0,s]=0;\\ d[0,t]=|(s,t)| &{} \text{ if } (s,t) \in E \text{ and } \text{ t } \text{ is } \text{ not } \text{ branching};\\ d[\beta ,t] = +\infty &{} \text{ if } d[\beta ,t] \text{ was } \text{ not } \text{ initialised}. \end{array} \end{aligned}$$

*Main recurrence:*

$$\begin{aligned} d[\beta ,t] = \left\{ \begin{array}{l} \min (\min _{v \in N^{-}(t)} \{d[\beta -1,v] + |(v,t)|\}, d[\beta -1,t]), \quad \text{ if } \text{ t } \text{ is } \text{ branching }\\ \min (\min _{v \in N^{-}(t)} \{d[\beta ,v] + |(v,t)|\}, d[\beta -1,t]), \quad \text{ if } \text{ t } \text{ is } \text{ not } \text{ branching. }\\ \end{array} \right. \end{aligned}$$

This recurrence works only on compressed graphs, i.e. it requires that the neighbours of simple vertices are branching. However, since the graph compression procedure described in “Preliminaries” section can be applied to general graphs, this recurrence is also applicable to general graphs. The calculation order for $d[\beta ,t]$ in the main recurrence must be by increasing value of $\beta$ and, for a fixed $\beta$, the branching vertices must be processed before the non-branching ones. Moreover, the shortest paths themselves can be constructed by a traceback procedure.

Finally, since the calculation of each value $d[\beta ,t]$ takes $O(|N^-(t)|)$ time, the algorithm runs in $O(b \sum _{t \in V}|N^-(t)|) =O(b |E|)$ time. We can guarantee that this algorithm runs in time polynomial in the length of the input by upper-bounding *b* by |*V*| (if $b > |V|$, we simply set $b=|V|$).□

As a corollary of Lemma 1, we can decide if $\mathcal {B}_{s}(\pi _1,\pi _2, G)$ is non-empty in *O*(*b*|*E*|) time. Now, using an argument similar to [1], i.e. the leaves of the recursion tree and the solutions are in one-to-one correspondence and the height of the recursion tree is bounded by 4*b*, we obtain the following theorem.

#### **Theorem 4**

*The* $(s,*,\alpha _1,\alpha _2,b)$-*bubbles can be enumerated in* $O(b^2|E||\mathcal {B}_s(G)|)$ *time. Moreover, the time elapsed between the output of any two consecutive solutions (i.e. the delay) is* $O(b^2|E|)$.

## Measuring the confidence of a transcript in full-length transcriptome assemblers

Reconstructing full-length transcripts from reads is a challenging task because two transcripts, even from different genes, may very well share subsequences that are longer than the sequenced reads, or even longer than the fragments in case of paired-end sequencing. This is specially true when genes host transposable elements within their introns, and less frequently but still present, within their UTRs and also exons (e.g. exonised repeats). Even if a repeat-containing intron is always spliced out in the splicing phase, this intron, and consequently the repeat, can still be present in RNA-seq data. The fraction of introns present in the sequenced data depends on the cell compartment that is sampled (nucleus, cytoplasm or both) and the protocol to remove rRNA (ribo-0 or polydT primers). As estimated in [9], the level of pre-mRNA can be assumed to vary between 2 and 22%. The true level of pre-mRNA may however be in practice higher, because the methods used for estimating it are mapping-based and therefore deal poorly with reads stemming from repeated regions. Besides, the upper bound given in [9] corresponds to extraction protocols which are harder to obtain. In this work, we considered the most commonly used extraction protocol to extract RNA, and assumed that they yielded pre-mRNA fractions between 5 and 15%. Thus, more introns than expected are sequenced, generating problems to transcriptome assemblers, particularly when they span several members of a specific repeat family.

**Fig. 4:**
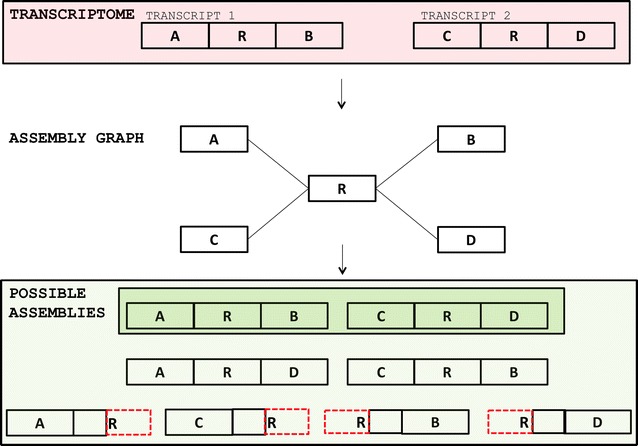
A theoretical scenario showing some problems repeats cause to assemblers. On the top of the figure, we can see two real transcripts containing each one a member of a repeat R. When building the assembly graph, the two copies of R may collapse into a single region of the graph, and connect the unique regions of both transcripts. The only correct assemblies are ARB and CRD, but the assembly graph also allows for the generation of the chimeric transcripts ARD and CRB, or truncated transcripts, in case the assembler chooses to be conservative

Most transcriptome assemblers are based on de Bruijn graphs and have no clear and explicit model for repeats in RNA-seq data, relying instead on heuristics to deal with them. Within the complex parts of the graph generated by repeats, any assembler will have to choose the “right” path(s) among the many present. Even with hints given by (paired-end) reads, assemblers can still have several arguable options to extend a contig (see Fig. 4). This problem gets harder if the (paired-end) reads do not span the repeat entirely, thereby not giving the assembler any reliable information on how to connect the unique regions. If the assembler decides to guess a path, it may erroneously extend a contig and create a chimeric transcript. It can also choose to be conservative by not choosing any path in complicated regions of the de Bruijn graph, and instead truncating the transcript. Although this strategy can lead to an accurate assembly, it will produce a very fragmented one, which is not desired. Whatever the strategy (conservative or permissive), the resulting assembled transcript may be erroneous (chimeric or truncated).

It is hence important to be able to identify low-confidence transcripts, which are the ones traversing complex regions of a de Bruijn graph, in order to know that the solution presented is the result of a “difficult” choice and therefore *may* not be the right one. To identify such transcripts, we introduce the concept of *Branching Measure* of a transcript. Consider the set of transcripts $\mathcal {T}$ output by a full-length transcriptome assembler starting from a set of reads $\mathcal {R}$. We construct the de Bruijn graph $G_k(R)$, and map back each transcript $t \in \mathcal {T}$ to the graph by identifying each of its *k*-mers. Given a positive integer *w*, let *W* be a *w*-sized window (or substring) with the largest number of branching *k*-mers in *t*. We define the Branching Measure of a transcript *t*, *B*(*t*), as the proportion of branching *k*-mers in *W*. By looking at *B*(*t*), it is possible to infer if *t* traversed a hard-to-assemble region in the de Bruijn graph, and this can be used as a measure of its confidence, i.e. the higher *B*(*t*) is, the lower is the confidence of *t*.

As a proof of concept, in the following we show two examples of the application of the Branching Measure to transcripts assembled by Trinity on RNA-seq data from the GEUVADIS project [18].

**Fig. 5:**
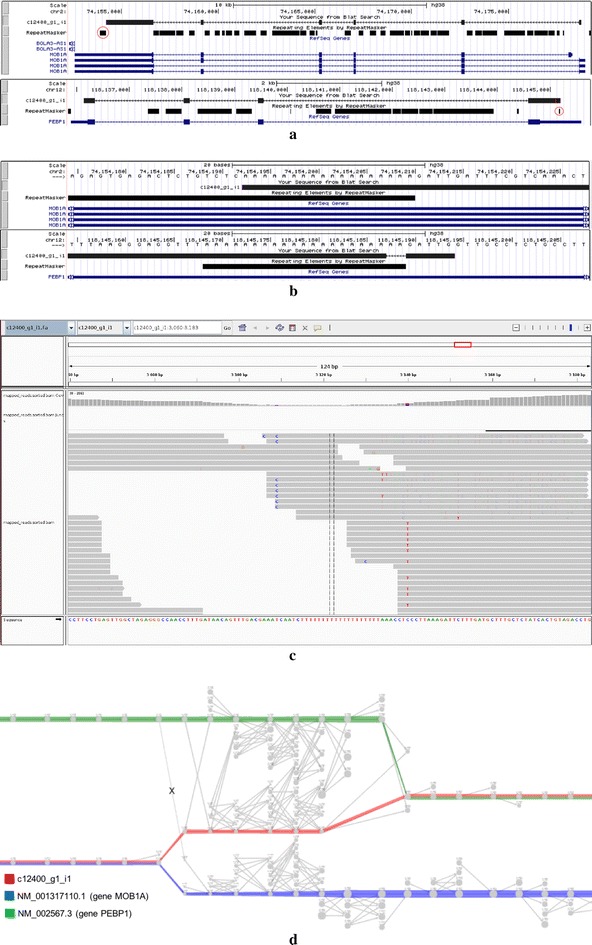
The chimeric transcript c12400_g1_i1 that aligns to the gene MOB1A in chromosome 2 and also to the gene PEBP1 in chromosome 12, in which the fusion of these genes is due to a small identical region shared between two different repeats present in their UTR regions (see “Measuring the confidence of a transcript in full-length transcriptome assemblers” section for details of each panel). **a** The alignment of the transcript c12400_g1_i1 to reference hg38, visualised using the UCSC Genome Browser. **b** The regions where both alignments intersect the repeats that cause the chimerism. **c** The mapping of all reads to transcript c12400_g1_i1 visualised using IGV. **d** A local visualisation of the subgraph induced by the *k*-mers of transcript c12400_g1_i1 at the junction point which causes the chimerism

 The first example (Fig. 5) is the chimeric transcript c12400_g1_i1 that aligns to the gene MOB1A in chromosome 2 and also to the gene PEBP1 in chromosome 12, in which the fusion of these genes is due to a small identical region shared between two different repeats present in their UTR regions. Figure 5a shows the alignment of the transcript c12400_g1_i1 to reference hg38, visualised using the UCSC Genome Browser. The alignment on the top shows that the built transcript aligns almost perfectly to an isoform of gene MOB1A in chromosome 2. Due to the repeats inside the red circles, the alignment is truncated in the 3′-UTR of MOB1A, and continued on the 5′-UTR of gene PEBP1 in chromosome 12 (alignment on the bottom). Thus, here we have a chimeric transcript. Figure 5b zooms in the regions where both alignments intersect the repeats that cause the chimerism. The main reason of the junction between the two genes is due to a stretch of 18 As shared between the A-tail of a SINE AluY in the 3′-UTR of MOB1A and a Simple Repeat A(n) in the 5′-UTR of PEBP1. Even though this repeated region is short, it was enough to cause problems to Trinity, which had access to 76-bp paired-end reads, with an average insert size of 158 bp. In Fig. 5c we mapped all reads back to transcript c12400_g1_i1 and visualised them using IGV [19]. As we can see, there are no single or paired-end reads traversing the small repeat. This shows that this chimera is not an in vitro or a biological one, but indeed an assembly mistake by Trinity. Figure 5d conveys a local visualisation of the subgraph induced by the *k*-mers of transcript c12400_g1_i1 at the junction point which causes the chimerism (the full graph can be accessed at http://kissplice.prabi.fr/bm/graph_chimera.html). We can see that this is a complex region since the transcript (red path) traverses a region having 11 branching *k*-mers in a window of 12, and could thus be flagged by the Branching Measure. There is no other such complex region in this transcript, i.e. this is the only hard-to-assemble region that this transcript goes through. We can also see in the picture the correct extension which should have been followed as the reference transcripts (the green and blue paths). Observe that even the reference transcripts could also have been flagged by our method since they traverse regions containing a concentration of branching vertices due to the repeated elements presented in Fig. 5a, b.

**Fig. 6:**
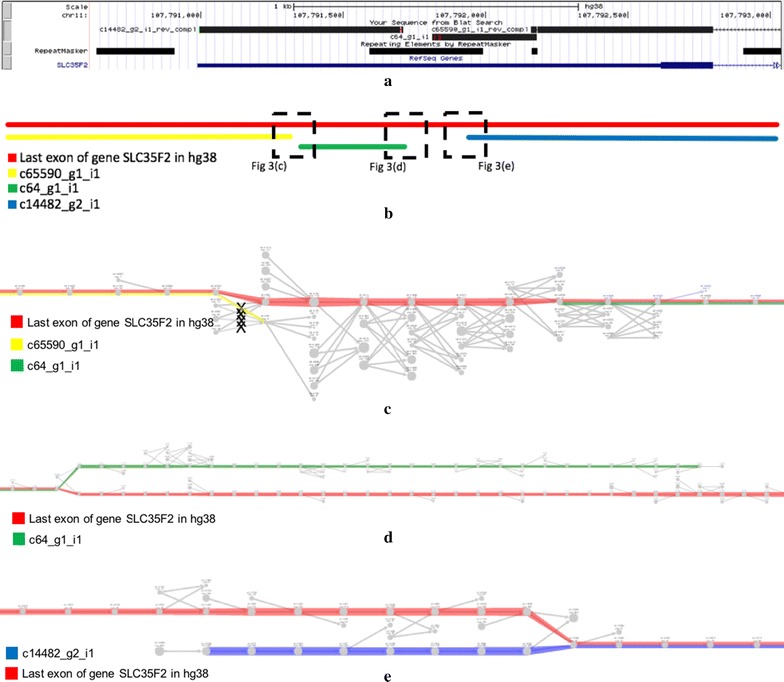
A mis-assembly of the last exon of gene SLC35F2, in which Trinity assembled several truncated transcripts instead of the full exon (see “Measuring the confidence of a transcript in full-length transcriptome assemblers” section for details of each panel). **a** The three truncated short transcripts: c65590_g1_i1, c64_g1_i1, and c14482_g2_i1. **b** A schematic global view on how the last exon of gene SLC35F2 was assembled by Trinity and how the three next figures are connected in the full graph drawing. **c** A local visualisation of the truncation point between c65590_g1_i1 and c64_g1_i1 due to a simple repeat. **d** A local view of the region that traverses the repeat AluJo, and where the assembler has chosen to truncate the transcript c64_g1_i1. **e** A local view of the region that traverses the repeat AluSz, and where the assembler has chosen to truncate the transcript c14482_g2_i1

The second case, depicted in Fig. 6, shows a mis-assembly of the last exon of gene SLC35F2, in which Trinity assembled several truncated transcripts instead of the full exon. Figure 6a shows, on the 3’ $\rightarrow$ 5’ orientation (reverse strand), the three truncated short transcripts: c65590_g1_i1, c64_g1_i1, and c14482_g2_i1. The truncation points were cause caused by repeats, where the first split is due to a simple repeat (A(n)) and the second is due to 2 consecutive Alus (AluJo and AluSz). Figure 6b displays a schematic global view on how the last exon of gene SLC35F2 was assembled by Trinity and how the three next figures are connected in the full graph drawing. This figure and the next assume the $5^{\prime }\rightarrow 3^{\prime }$ orientation. Figure 6c conveys a local visualisation of the truncation point between c65590_g1_i1 and c64_g1_i1 due to a simple repeat. We can see that Trinity mis-assembled the very end of c65590_g1_i1 (only the last base) and truncated the transcript. The yellow path is accurate although truncated and does not go through a complicated region (one having a concentration of branching vertices). Even though the reference exon path in this region has 11 consecutive branching vertices and would be flagged by the Branching Measure, this method is unable to flag c65590_g1_i1 since it is truncated too early, before entering the complex region. Figure 6d shows a local view of the region that traverses the repeat AluJo, and where the assembler has chosen to truncate the transcript c64_g1_i1. We can see that Trinity mis-assembled the last 29 bases of c64_g1_i1 and truncated it. At the end of c64_g1_i1, we have 23 branching vertices in a window of 34 vertices, so this truncated transcript can be flagged by our method, as it is deeply enough plunged into a complex region. Finally, Fig. 6e displays a local view of the region that traverses the repeat AluSz, and where the assembler has chosen to truncate the transcript c14482_g2_i1. Again, the Branching Measure is not able to flag this transcript since it is not deeply enough plunged into a complex region. The full graph of Fig. 6b–e can be accessed at http://kissplice.prabi.fr/bm/graph_truncated.html.

## Experimental results

### Local assembly: experimental setup

To evaluate the performance of our method, we simulated RNA-seq data using the FluxSimulator version 1.2.1 [20]. We generated 100 million reads of 75 bp using its default error model. We used the RefSeq annotated Human transcriptome (hg19 coordinates) as a reference and we performed a two-step pipeline to obtain a mixture of mRNA and pre-mRNA (i.e. with introns not yet spliced). To achieve this, we first ran the FluxSimulator with the Refseq annotations. We then modified the annotations to include the introns and re-ran it on this modified version. In this second run, we additionally constrained the expression values of the pre-mRNAs to be correlated to the expression values of their corresponding mRNAs, as simulated in the first run. Finally, we mixed the two sets of reads to obtain a total of 100M reads. We tested two values, namely 5 and 15% for the proportion of reads from pre-mRNAs. Those values were chosen so as to correspond to realistic ones (see “Measuring the confidence of a transcript in full-length transcriptome assemblers” section).

On these simulated datasets, we ran KisSplice [12] versions 2.1.0 (Ks_2.1.0) and 2.2.0 (Ks_2.2.0, with a maximum number of branching vertices set to 5) and obtained lists of detected bubbles that are putative alternative splicing (AS) events. We also ran the full-length transcriptome assemblers Trinity version r2013_08_14 and Oases version 0.2.09 on both datasets, obtaining a list of predicted transcripts, from which we then extracted a list of putative AS events. For Oases, there was one locus in each dataset for which we were not able to extract the putative AS events. A manual inspection revealed that they were mostly composed of subparts of introns or UTRs flanked by repeats, usually copies of ALUs. The presence of such high copy-number repeats in these transcripts induced the clustering of all these unrelated sequences into one complex locus. More precisely, in the dataset containing 5% of the reads from pre-mRNAs, the largest locus that Oases predicted comprised 20,769 transcripts, while the second largest contained only 139 transcripts. In the other simulated dataset, the largest locus comprised 39,389 transcripts, and the second largest contained only 205 transcripts. This indicates that Oases faces similar issues to Ks_2.1.0. For fairness of comparison, we did not post-process these complex loci, and we were then unable to retrieve the potential AS events that they could describe. It is worth mentioning though, that the majority of the transcripts inside these loci corresponded to subparts of introns or UTRs, and not to full introns or exons, and therefore could not describe AS events.

In order to assess the precision and the sensitivity of our method, we compared our set of *found* AS events to the set of *true* AS events. Following the definition of Astalavista, an AS event is composed of two sets of transcripts, the inclusion/exclusion isoforms respectively. We consider that an AS event is *true* if at least one transcript among the inclusion isoforms and one among the exclusion isoforms is present in the simulated dataset with at least 5 reads per kilobase (RPK). The rationale for adding this threshold is that very rare events are considerably hard, or even impossible, to detect by all methods.

To compare the results of KisSplice with the *true* AS events, we propose that a true AS event is a *true positive* (TP) if there is a bubble such that one path matches the inclusion isoform and the other the exclusion isoform. If there is no such bubble among the results of KisSplice, the event is counted as a *false negative* (FN). If a bubble does not correspond to any *true* AS event, it is counted as a *false positive* (FP). To align the paths of the bubbles to transcript sequences, we used the Blat aligner [21] with 95% identity and a constraint of 95% of each bubble path length to be aligned (to account for the sequencing errors simulated by FluxSimulator). We computed the sensitivity TP/(TP+FN) and precision TP/(TP+FP) for each simulation case and we report their values for various classes of expression of the minor isoform. Expression values are measured in RPK.

### Local assembly: results

**Fig. 7:**
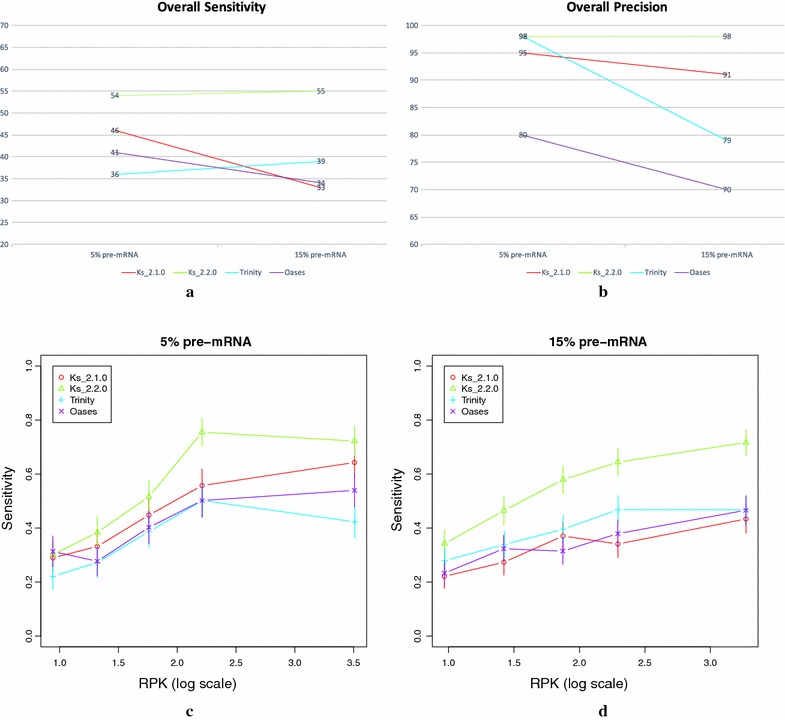
The overall values for sensitivity and precision, and the detailed sensitivity by expression levels of Ks_2.1.0, Ks_2.2.0, Trinity and Oases on the two simulated datasets. **a** Overall sensitivity of the four methods on the two simulated datasets. **b** Overall precision of the four methods on the two simulated datasets. **c** Detailed sensitivity by expression levels of the four methods on the 5% pre-mRNA dataset. **d** Detailed sensitivity by expression levels of the four methods on the 15% pre-mRNA dataset. The expression levels in **c** and **d** represent several classes of expression of the minor isoform. Each class (i.e. point in the graph) contains the same number of AS events. It is therefore an average sensitivity on a potentially broad class of expression

The overall sensitivity and precision of Ks_2.2.0, Ks_2.1.0, Trinity and Oases can be found in Fig. 7a, b, respectively. A first look reveals that Ks_2.2.0 outperforms the other three methods in both measures and datasets.

A closer look at Fig. 7a shows that both versions of KisSplice had better sensitivity than both transcriptome assemblers in the 5% pre-mRNA dataset. However, due to its inability to deal with repeat-associated regions, the performance of Ks_2.1.0 drops substantially, from 46 to 33%, when a higher rate of 15% of pre-mRNA is present in the data. The same happened with Oases. Ks_2.2.0 and Trinity, on the other hand, were able to slightly improve their sensitivity from the 5 to the 15% pre-mRNA dataset. It is however worth mentioning that the sensitivity of Ks_2.2.0 is substantially higher than the one of Trinity in the 15% pre-mRNA dataset. In summary, we can say that Ks_2.2.0 is substantially more sensitive than all the other three methods. This reflects the fact that most problematic repeats are in introns. A small unspliced mRNA rate leads to few repeat-associated subgraphs, so there are not many AS events drowned in them. In this case, the advantage of using Ks_2.2.0 is less obvious, whereas a large proportion of pre-mRNA leads to more AS events drowned in repeat-associated subgraphs which are identified by Ks_2.2.0 and usually missed by the other methods.

In Fig. 7b we can see that Ks_2.2.0 and Trinity presented the highest precision (98%) of all methods in the 5% pre-mRNA dataset. Although Ks_2.1.0 is ranked third, it still presents a very high precision (95%), while Oases presented a moderate value (80%). Nevertheless, the most important aspect to be observed in Fig. 7b is that Ks_2.2.0 kept the same high precision even when a higher rate of 15% of pre-mRNA is present in the data. Trinity, on the other hand, dropped significantly from 98 to 79%. This drop in precision is actually mostly due to the prediction of a large number of intron retentions, since Trinity assembles both the mRNA and the pre-mRNA. Ks_2.1.0 dropped slightly from 95 to 91%, and Oases dropped moderately, from 80 to 70%. In summary, we can say that both versions of KisSplice are more precise than both transcriptome assemblers, except that Trinity shows comparable precision if a small rate of pre-mRNA is present in the data, and, more specifically, that Ks_2.2.0 outperformed all the other three methods. The high precision we obtain indicates that we have very few false positives. Those mostly correspond to repeat-induced bubbles mistakenly identified as AS events.

Finally, Fig. 7c, d present the detailed sensitivity by expression levels of the four methods on both datasets, allowing for a better understanding of their performance. As we can see, Ks_2.2.0 presented the best sensitivity in practically all expression levels in both datasets, while the other three methods were worse, but comparable between themselves. We can also observe that the gap between the sensitivity of Ks_2.2.0 and the sensitivity of the other methods tends to increase as the expression levels of the genes increase, especially in the 15% pre-mRNA dataset. Since highly-expressed genes tend to present higher levels of pre-mRNA content, they bring several repeat copies in their introns, and thus some parts of their associated graphs are complex and repeat-induced. Therefore, AS events inside such genes tend to be trapped in troublesome regions of the graph, making them harder to find. As Ks_2.2.0 is able to avoid such complex regions and retrieve the AS events deeply plunged into them, it presents better sensitivity than the other methods, especially in highly-expressed genes and datasets with high rate of pre-mRNAs.

As was already reported in [12], KisSplice (i.e. both Ks_2.2.0 and Ks_2.1.0) is faster and uses considerably less memory than Trinity and Oases. For instance, on these datasets, KisSplice uses around 5 GB of RAM, while Trinity uses more than 20 GB, and Oases, around 18 GB. However, it should be noted that both these latter methods try to solve a more general problem than KisSplice, that is reconstructing the full-length transcripts.

To conclude, our results show that Ks_2.2.0 is significantly more sensitive and precise than Ks_2.1.0, Trinity and Oases for the task of identifying AS events. The advantage of using Ks_2.2.0 over the other three methods is more evident when the input data contains high pre-mRNA content or the AS events of interest stem from highly-expressed genes.

### On the usefulness of Ks_2.2.0 on real data

**Fig. 8:**
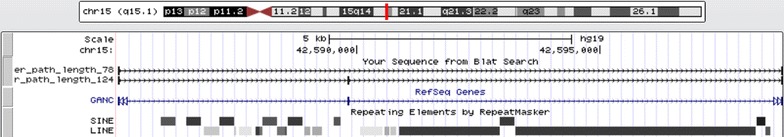
One of the bubbles found only by Ks_2.2.0 with the corresponding sequences mapped to the reference human genome and visualised using the UCSC Genome Browser. The *first two lines* correspond to the sequences of, respectively, the shortest (exon exclusion variant) and longest paths (exon inclusion variant) of the bubble mapped to the genome. The *blue line* is the Refseq annotation. The *last line* shows the annotated SINE and LINE sequences (transposable elements)

In order to give an indication of the usefulness of our repeat-avoiding bubble enumeration algorithm with real data, we also ran Ks_2.2.0 and Ks_2.1.0 on the SK-N-SH Human neuroblastoma cell line RNA-seq dataset (wgEncodeEH000169, total RNA). In Fig. 8, we have an example of a *non-annotated* exon skipping event not found by Ks_2.1.0. Observe that the intronic region contains several transposable elements (many of which are Alu sequences), while the exons contain none. This is a good example of a bubble (exon skipping event) drowned in a complex region of the de Bruijn graph. The bubble (composed by the two alternative paths) itself contains no repeated elements, but is surrounded by them. In other words, this is a bubble with few branching vertices that is surrounded by repeat-associated subgraphs. Since Ks_2.1.0 is unable to differentiate between repeat-associated subgraphs and the bubble, it spends a prohibitive amount of time in the repeat-associated subgraph and fails to find the bubble.

### Global assembly

To test our hypothesis that the Branching Measure is able to identify problematic transcripts, we evaluated it on the transcripts assembled by Trinity on the two simulated RNA-seq datasets described in “Local assembly: results” section, and on two other real RNA-seq datasets: one from the GEUVADIS project [18]^1^ and one from a neuroblastoma SK-N-SH cell line (ENCODE) differentiated or not using retinoic acid.^2^ Even though our method is reference-free, we have chosen to evaluate it under a model species so that we could make use of annotated reference genomes to assess if our predictions are correct. We compared our measure against two state-of-the-art methods for de novo transcriptome evaluation, Rsem-Eval [4] and TransRate [5], on the specific task of identifying chimeric transcripts in Trinity’s assemblies on all four described datasets. In all our tests, we used the *contig impact score* of Rsem-Eval as a measure of contig correctness. Formally, the *contig impact score* is a statistical measure that compares the hypothesis that a particular contig (i.e. transcript) is a true contig with the null hypothesis that the reads composing the contig actually represent the background noise [4]. In other words, the *contig impact score* determines the relative contribution of each transcript to explaining the assembly. Well-assembled transcripts should therefore have a high *contig impact score*, and badly assembled transcripts, including chimeras, should have a low *contig impact score*. TransRate [5], on the other hand, allowed us to work with a specific metric representing the probability that a contig is derived from a single transcript. This metric denotes the probability that the read coverage of a transcript is best modelled by a single Dirichlet distribution, rather than two or more distributions, and it corresponds to the field sCseq of TransRate’s output file contigs.csv.

**Fig. 9:**
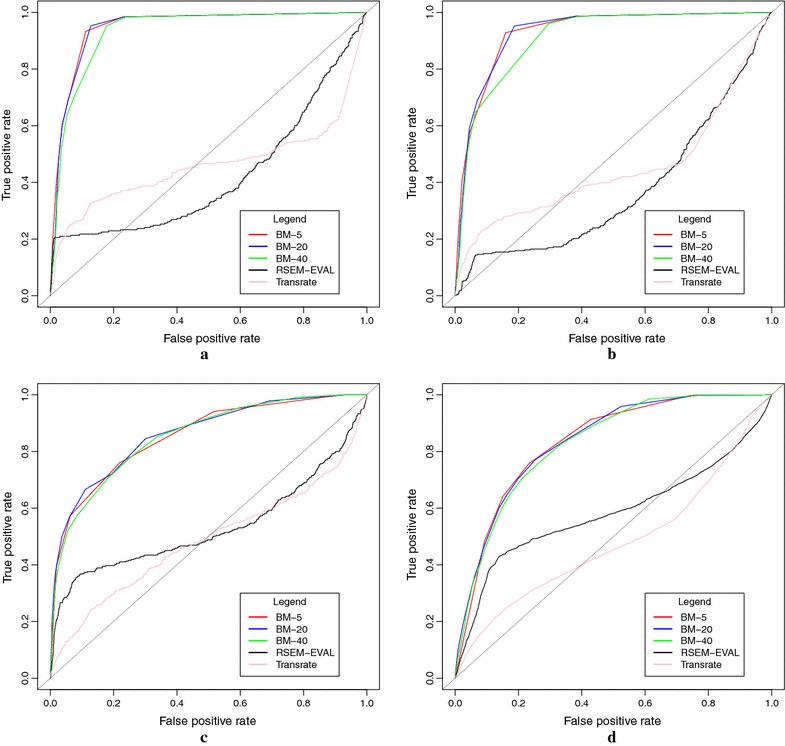
The performance of the Branching Measure, Rsem-Eval, and TransRate on identifying chimeric transcripts on the four datasets described in “Global assembly” section. BM-*x* stands for Branching Measure using a window of size *x*. In this test, the 10% leftmost and rightmost parts of the transcripts were disregarded in the Branching Measure calculation. **a** Simulated dataset with 5% pre-mRNA. **b** Simulated dataset with 15% pre-mRNA. **c** GEUVADIS dataset. **d** SKNSH dataset


As was shown before, one of the main errors that transcriptome assemblers do is to build chimeric transcripts. We compared the performances of the Branching Measure, Rsem-Eval, and TransRate on identifying chimeric transcripts. In order to have our ground truth, we first identified which assembled transcripts are chimeric with respect to a reference genome by using Algorithm 1. In total, 253 out of 18,706 transcripts (1.3%) in the 5% pre-mRNA dataset, 376 out of 26,407 transcripts (1.4%) in the 15% pre-mRNA dataset, 375 out of 99,591 transcripts (0.3%) in the GEUVADIS dataset, and 2830 out of 457,383 transcripts (0.6%) in the SKNSH dataset were classified as chimeric. Figure 9 depicts four ROC curves showing the performance of the three methods on all datasets. We can observe that the Branching Measure outperforms both Rsem-Eval and TransRate by a large margin in all tests and, with a high-value threshold, is also able to flag a majority of the chimeric transcripts while keeping a low false positive rate. These experiments show that, in the provided datasets, chimeric transcripts could be well captured by the Branching Measure. Our false positives include well-assembled transcripts traversing high copy-number low divergence repeats, and our false negatives include chimeric transcripts that did not go through a complex region. The main issue with Rsem-Eval and TransRate, on the other hand, is that both methods failed to find chimeric transcripts assembled from genes with similar expression levels. These chimeras had low scores and corresponded to the false negatives at the end of the ROC curves for Rsem-Eval and TransRate. As a side effect of this misclassification, many well-assembled transcripts had higher scores than several real chimeras, and were mistakenly flagged as chimeras.

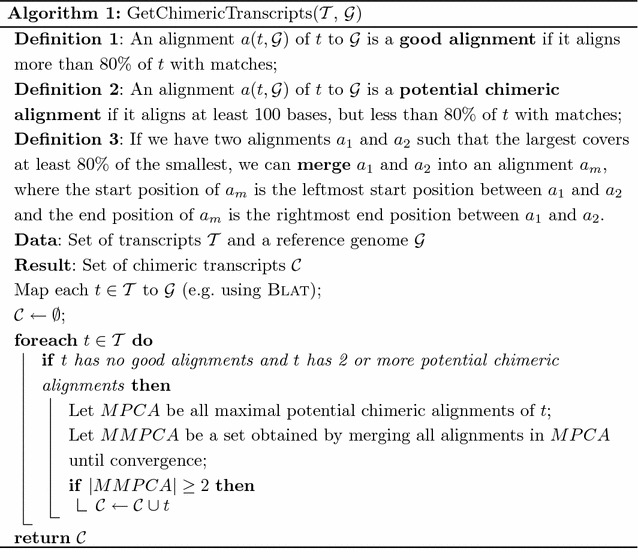


## Concluding remarks and perspectives

Although transcriptome assemblers are now commonly used, their way to handle repeats is not satisfactory, arguably because the presence of repeats in transcriptomes has been underestimated so far. Given that most RNA-seq datasets correspond to total mRNA extractions, many introns are still present in the data and their repeat content cannot be simply ignored. Although repeats in transcriptomic and genomic data cause similar problems to assemblers, the specificities of each mean that a successful strategy in one context may fail in the other. It is thus essential for transcriptome assemblers to formally address the repeats problem.

In this paper, we first proposed a simple formal model for representing high copy-number repeats in RNA-seq data. Exploiting the properties of this model, we established that the number of compressible arcs is a relevant quantitative characteristic of repeat-associated subgraphs. We proved that the problem of identifying in a de Bruijn graph a subgraph with this characteristic is NP-complete. However, this characteristic drove the design of an algorithm for efficiently identifying AS events that are not included in repeated regions. The new algorithm was implemented in KisSplice version 2.2.0, and by using simulated RNA-seq data, we showed that it improves significantly the sensitivity of the previous version of KisSplice, while also improving its precision. In addition, we compared our algorithm to Trinity and Oases, and showed that for the specific task of calling AS events, our algorithm is significantly more sensitive while also being more precise. Our results also showed that the advantage of using KisSplice version 2.2.0 is more evident when the input data contains high pre-mRNA content or the AS events of interest stem from highly-expressed genes. Moreover, we gave an indication of the usefulness of our method on real data. Finally, we explored the proposed model in the context of full-length transcriptome assembly by introducing the Branching Measure, which is able to flag the transcripts that go through a complex region in the de Bruijn graph. Even though one should not directly consider low-confidence transcripts as erroneous ones since they could have been correctly assembled despite the hardness, the described measure is useful from a user’s point-of-view since it enables to flag the transcripts that result from a “difficult” choice during the assembly, no matter which assembler is used. We showed that this measure can indeed capture assembly errors in real cases and, when compared to Rsem-Eval [4] and to TransRate [5] on the specific task of identifying chimeric transcripts, the measure we propose outperformed the ones used by Rsem-Eval and TransRate by a large margin. The originality of our work, when compared to other methods for transcriptome assembly evaluation, is that we use only the topology of the graph. Despite the successful application of the Branching Measure in global transcriptome assembly, it remains a simple method, and in particular, we would like to emphasise that it must be seen as a proof of concept that exploring the topology of the subgraph around a transcript can give some hints about its confidence level, quality, assembly hardness, etc. The method proposed is not a full-fledged one for assessing transcripts in a de novo context. It could however be a promising direction to improve transcriptome assembly evaluation, especially when combined with statistical and read-mapping approaches (e.g. Rsem-Eval [4] or TransRate [5]).

As concerns the local transcriptome assembly of variations, the most interesting open problem which currently remains is how to efficiently enumerate AS events whose variable region (e.g. skipped exon, retained intron) traverses repeats. Although the application of the proposed model enabled to retrieve several AS events that were previously missed, the current algorithm is still only able to *avoid* repeats, not to solve them. The presence of repeats in RNA-seq data shows that transcriptome assemblers should formally address the repeats issue, as is generally the case of genome assemblers, preferably by solving them. Even if repeats are less frequent in transcriptomic data and are thus easier to solve than in the genomic context, the complexity and ambiguity they add are enough to cause problems if not addressed properly. If this is not done, it will impact the assembly of full-length transcripts or variants, leading to either erroneous or fragmented ones, especially in regions that are more prone to contain repeats, such as introns, UTRs, and exonised repeats.

As concerns future works, our repeats model could be improved. One direction would be to employ a tree-like structure to take into account the evolutionary nature of repeat (sub)families. Variability in the sizes of the copies of a repeat family would also enable to model more realistically the true nature of families of transposable elements (the type of repeats which cause most trouble in assembly). Another example would be to explicitly model sequencing errors in Theorem 1. Although, in practice, assemblers like KisSplice [1] employ a sequencing error removal module, it remains unclear how to distinguish the structures created by sequencing errors from the ones induced by a lowly-expressed member of a highly-expressed family of repeats, or by infrequent allelic differences in pool-seq. The difficulty increases in regions that are highly expressed or that present sequencing error bias. In practice, error removal strategies may be too stringent and erroneously remove SNPs and repeats. Taking into account the sequencing errors in the model would make it applicable even without any pre-processing of the data, and would thus be more sensitive for finding repeats if such errors are correctly modeled. Finally, the Branching Measure could also be extended to identify truncated transcripts and isoforms stemming from paralogous genes.

## Footnotes

1 This dataset can be found at the ArrayExpress database (http://www.ebi.ac.uk/arrayexpress/) under the accession number E-GEUV-6, and we used the individual named NA06994, extract name “NA06994.2.M_111215_7 extract”.

2 This dataset can be found at http://genome.crg.es/encode_RNA_dashboard/hg19/, and is also accessible with the following accession numbers: ENCSR000CPN—SRA: SRR315315, SRR315316 and ENCSR000CTT—SRA: SRR534309, SRR534310. For cell lines treated by retinoic acid, the reads were 76nt long, while they were 100nt long for the non treated cells. Hence we trimmed all reads to 76nt.
